# Deficiency of circadian protein CLOCK reduces lifespan and increases age-related cataract development in mice

**DOI:** 10.18632/aging.100241

**Published:** 2010-12-09

**Authors:** Yuliya V. Dubrovsky, William E. Samsa, Roman V. Kondratov

**Affiliations:** Department of Biological, Geological and Environmental Sciences and Center for Gene Regulation in Health and Disease, Cleveland State University, Cleveland, OH 44115, USA

**Keywords:** dermatitis, circadian rhythms, aging, transcription

## Abstract

Circadian clock is implicated in the regulation of aging. The transcription factor CLOCK, a core component of the circadian system, operates in complex with another circadian clock protein BMAL1. Recently it was demonstrated that BMAL1 deficiency results in premature aging in mice. Here we investigate the aging of mice deficient for CLOCK protein. Deficiency of the CLOCK protein significantly affects longevity: the average lifespan of *Clock^−/−^* mice is reduced by 15% compared with wild type mice, while maximum lifespan is reduced by more than 20%. CLOCK deficiency also results in the development of two age-specific pathologies in these mice, cataracts and dermatitis, at a much higher rate than in wild type mice. In contrast to BMAL1 deficient animals, *Clock^−/−^* mice do not develop a premature aging phenotype and do not develop the multiple age-associated pathologies characteristic of BMAL1 deficiency. Thus, although CLOCK and BMAL1 form a transcriptional complex, the physiological result of their deficiency is different. Our results suggest that CLOCK plays an important role in aging, specifically; CLOCK activity is critical for the regulation of normal physiology and aging of the lens and skin.

## INTRODUCTION

The circadian clock is a genetically determined timekeeping system important for synchronization of an organism's everyday activity with the periodic light/darkness changes in the environment. According to the current paradigm, the mammalian circadian clock is organized in a hierarchical way: the master clock located in the suprachiasmatic nucleus (SCN) of the anterior hypothalamus is regulated by signals from the retina, and in turn synchronizes so called peripheral oscillators located in most organs and tissues [1]. These peripheral oscillators are responsible for the generation of rhythms in physiology [2,3]. Many physiological processes such as metabolism, hormone production and the sleep-wake cycle are under direct control of the circadian clock [4,5]. The importance of the circadian clock for human physiology is demonstrated by multiple reports that link disturbances in circadian oscillations to different pathologies in humans [4,6]. Recently, connections between the circadian clock and aging have been established through the analysis of aging in model organisms with targeted disruptions or mutations in different circadian clock genes [7].

BMAL1 (Brain and Muscle ARNT Like protein 1, also known as MOP3, ARNTL, and ARNT3), is a core component of the circadian system; deficiency of BMAL1 leads to the disturbance of circadian behavior and disruption of normal circadian patterns in gene expression in the brain and peripheral tissues such as the liver, kidney and muscles [8]. BMAL1 activity is implicated in the control of aging; indeed, mice deficient for BMAL1 have a reduced lifespan and develop multiple age-associated pathologies: a phenotype which can be described as accelerated aging [9,10]. BMAL1 is a basic helix-loop-helix (bHLH) PAS domain containing transcriptional factor, which forms a complex with another bHLH-PAS domain containing transcriptional factor CLOCK [11,12] (or in some tissues with the CLOCK paralog NPAS2 [13,14]). The BMAL1:CLOCK or BMAL1:NPAS2 complexes regulate the expression of the circadian clock and clock output genes [15].

CLOCK was recently linked to aging induced by non-lethal irradiation in mice [16] In accordance with its central role in the circadian system, mice with a mutation in the *Clock* gene lose circadian behavior and have a disrupted pattern of circadian gene expression [17,18]. Surprisingly, mice with a targeted disruption of the *Clock* gene demonstrate nearly normal circadian behavior [19], most likely because NPAS2 functionally substitutes for CLOCK activity in the SCN [20] in these knock-out mice. The difference between *Clock* mutation and *Clock* gene disruption (knockout) is that with *Clock* mutation, the non-functional CLOCK protein may compete with NPAS2 for interaction with BMAL1, producing a transcriptionally inactive complex [20-22]. Here we investigate the aging of CLOCK deficient mice in order to better understand the role of this protein and the circadian clock in aging.

## RESULTS

**Figure 1.**
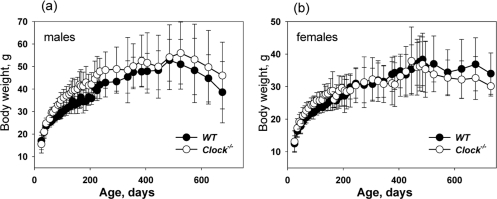
CLOCK deficiency does not affect body weight of mice. Age-dependent changes in body weight of wild type and *Clock^−/−^* mice. (**a**) Males, (**b**) females.

*Clock^−/−^* mice were born with expected ratio and developed normally; we did not detect any difference between young wild type and *Clock^−/−^* mice. In order to investigate the role of the CLOCK protein and peripheral circadian oscillation in aging, we monitored normal aging of mice with a targeted disruption of the *Clock* gene and compared it with the premature aging phenotype of BMAL1 deficient mice. As previously reported [9], *Bmal1^−/−^* mice demonstrate growth retardation at 3-4 months of age, reach maximum weight at around 4-6 months of age and then start losing weight. In sharp contrast to *Bmal1^−/−^* mice, there was no statistically significant difference between weight accumulation of *Clock^−/−^* and wild type mice (Figure 1a and 1b). It was reported that the weight of *Clock/Clock* mutant mice was significantly higher than the weight of wild type mice, most likely as a consequence of hyperphagia [23]. In contrast to that, *Clock^−/−^* mice did not demonstrate increased weight; thus, *Clock* mutation and deficiency have different effects on weight accumulation in mice. We did observe some weight increase for *Clock^−/−^* males compared with wild type males, but the difference was not statistically significant. The reason for this difference is unclear; one possible explanation is that in *Clock/Clock* mutants the CLOCK protein is present but its transcriptional activity is disrupted, while in *Clock^−/−^* mice, the CLOCK protein is absent. It was suggested that mutant CLOCK may act as a dominant negative protein [19,22].

**Figure 2.**
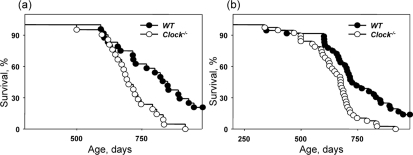
Effect of CLOCK on mouse lifespan. Kaplan-Meyer survival curves of wild type and *Clock^−/−^* mice of both genders (**a**) Survival data for death only from natural causes, (**b**) combined data for mice which died from natural causes and mice sacrificed due to severe dermatitis. For both graphs, the genotypes differ significantly (p<0.01).

*Bmal1^−/−^* mice have a significantly reduced lifespan (about 8 months on average [9]). CLOCK deficiency does not give rise to such a dramatic effect in the lifespan of mice. Figure 2 represents the Kaplan-Meyer survival curves of wild type and *Clock^−/−^* mice. We did not detect any significant difference between the lifespans of males and females; therefore, data for both genders were combined. Many *Clock^−/−^* mice developed dermatitis (see later). Animals with severe dermatitis were euthanized; thus, Figure 2a represents survival curves of mice that died by natural causes, while 2b represents combined data including euthanization. The average lifespan of mice that died from natural causes (Figure 2a) is 705.5 +/− 94.7 days for *Clock^−/−^* mice and more than 808.3 +/− 132.2 days for wild type mice (5 mice are still alive). The average lifespan of mice that died from both natural causes and were sacrificed due to development of severe dermatitis (Figure 2b) is 676.6 +/− 115.4 days for *Clock^−/−^* mice and more than 736.3 +/− 162.3 days for wild type mice. Thus, a deficiency of the CLOCK protein reduces average lifespan in mice. The effect of a CLOCK deficiency on maximum lifespan was even more dramatic: only 19% of *Clock^−/−^* mice survived after 800 days and only 1 mouse survived after 900 days, while for wild type mice, 58% of animals survived after 800 days and 29% of mice survived after 900 days, with 5 wild type mice currently (at the time of submission of this paper) alive.

**Figure 3.**
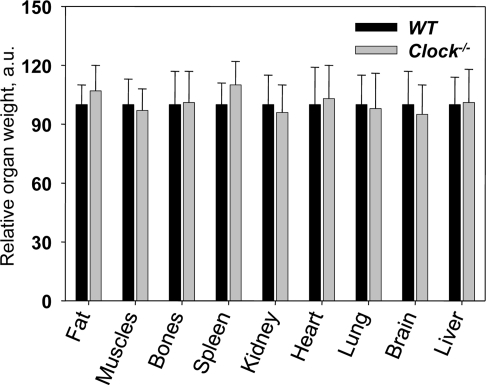
Relative weights of major organs from 24-months old wild type and *Clock^−/−^* mice. Relative weights were calculated as the organ weight divided by body weight, and the average value for wild-type mice for each organ was set to 100.

*Bmal1^−/−^* mice demonstrate the development of multiple age-associated pathologies such as cataracts, sarcopenia, reduced hair growth and progressive atrophy of internal organs: the weight of the spleen, muscles and kidneys is significantly reduced in aged *Bmal1^−/−^*mice [9]. We compared the weight of the same organs between wild type and *Clock^−/−^* mice at different ages. We did not detect any difference in organ weight between genotypes neither for young (data not shown) nor for old mice (Figure 3). We also did not observe any gross or histological differences. Thus, deficiency of CLOCK does not result in any significant age-associated changes in the organs and tissues which are affected in the *Bmal1^−/−^* mice. The difference between CLOCK and BMAL1 deficient mice suggests that either BMAL1 and CLOCK have different roles in the regulation of physiology, or that CLOCK is functionally substituted by NPAS2 or another protein.

**Figure 4.**
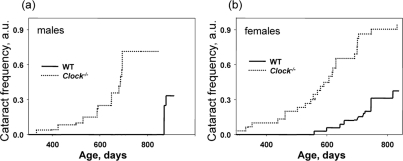
Deficiency of CLOCK results in increased rate of cataract development. Chronological incidence of cataracts in wild type and Clock^−/−^ mice. Abscissas show the mouse ages at which data for each time point were collected. Ordinates show the fraction of eyes with cataracts. (**a**) Males, (**b**) females. For both graphs, the genotypes differ significantly (p<0.01).

The only pathology which was a feature of the *Bmal1^−/−^* mice and also developed in *Clock^−/−^* mice were cataracts. Figure 4a and b shows the time of cataract development in wild type and *Clock^−/−^* mice. For *Clock^−/−^* mice, we detected the first cataract as early as at 330 days of age for females and 390 days for males; more than 70% of males and more than 90% females developed cataracts at old age (older than 800 days). In sharp contrast, in wild type mice the first cataract was detected at around 600 days of age for females and after 800 days for males, and only 30% of animals developed cataracts at old age. Thus, deficiency of CLOCK results in the early development of cataracts in mice. In strong agreement with these data, mice with a mutation in the *Clock* gene also demonstrated significantly increased level of cataract development after non-lethal irradiation. Very similar results on cataract development were obtained for *Bmal1^−/−^*mice. BMAL1 and CLOCK form a transcriptional complex, therefore, together these data on cataract development strongly suggest that BMAL1:CLOCK transcriptional activity plays an important role in normal lens physiology and can be a target for the development of therapy for cataract treatment and prevention.

**Figure 5.**
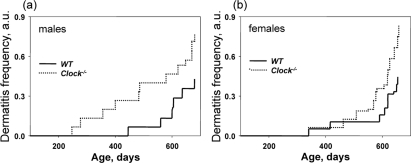
Deficiency of CLOCK results in an increased rate of dermatitis. Chronological incidence of dermatitis in wild type and *Clock^−/−^* mice. Abscissas show the mouse ages at which data for each time point were collected. Ordinates show the fraction of mice with detected dermatitis. (**a**) Males, (**b**) females. For both graphs, the genotypes differ significantly (p<0.01).

Dermatitis is common in C57B6 mice but the cause appears to be nonspecific. We noticed that *Clock^−/−^* mice developed dermatitis at a higher frequency than wild type mice; therefore, we decided to carefully monitor dermatitis development similarly to the development of cataracts. In the majority of the cases, dermatitis was mild, but for 38% of *Clock^−/−^* mice and 31% of wild type mice it led to the formation of ulcers, and those animals had to be euthanized. Figure 5 represents data on the incidence of dermatitis in wild type and *Clock^−/−^* mice. Interestingly, the difference in the development of dermatitis between genotypes was more obvious for males (Figure 5a) than for females (Figure 5b), which may be caused by frequent fighting between males (males of C57B6 background are highly aggressive). At this time we cannot identify the cause of dermatitis in *Clock^−/−^* mice, but our data suggests that CLOCK and probably other circadian proteins play an important role in the aging of skin and support further studies on this subject.

## DISCUSSION

The circadian clock regulates multiple physiological processes^5^. Recent data obtained in model organisms link the circadian clock with aging: in mice, deficiency of BMAL1 results in accelerated aging [9,10], while deficiency of PER1 and PER2 and a mutation in CLOCK leads to the development of premature aging only after exposure to non-lethal doses of gamma radiation [16,24]. In *Drosophila melanogaster*, a null mutation in the circadian clock gene *period* leads to accelerated aging [25], whereas increased expression of *takeout* extends the life span [26]. The fact that several different components of the circadian clock are involved in the regulation of aging supports the idea that an intact circadian clock is important for longevity, and disruption of circadian oscillations may lead to the acceleration of aging, probably through disruption of the synchronization of metabolic processes [7].

In this report we investigate the effect of a deficiency of the circadian protein CLOCK on aging. Transcriptional factor CLOCK was the first mammalian circadian clock gene discovered; a mutation in *Clock* results in the loss of circadian behavior [17]. Surprisingly, targeted disruption of *Clock* did not influence circadian locomotor activity, but did affect the circadian profile of gene expression in peripheral tissues [19,21]. As demonstrated later, NPAS2 functionally substitutes for CLOCK in the suprachiasmatic nucleus (SCN) of the anterior hypothalamus (the site of the master circadian clock), which provides an explanation for circadian rhythms in locomotor activity, but NPAS2 cannot compensate for the absence of CLOCK in peripheral tissues [20,21]. In agreement with functional redundancy of NPAS2 and CLOCK [20,27], only double deficiency of CLOCK and NPAS2 in mice results in arthropathy [28](E.A. Yu and D.R. Weaver, personal communication) similar to arthropathy developed upon BMAL1 deficiency [10] CLOCK is the major interacting partner of BMAL1 [12]. The BMAL1:CLOCK transcriptional complex may function as a transactivator or transrepressor [29] depending on its interaction with the circadian proteins CRY1 and CRY2. Consistent with these proteins being major partners of each other, the expression of BMAL1:CLOCK target genes is significantly affected in the tissues of both *Clock^−/−^* and *Bmal1^−/−^* mice [8,19].

CLOCK deficiency does not affect mouse development; young *Clock^−/−^* mice are indistinguishable from wild type littermates. In contrast to *Bmal1^−/−^* mice, *Clock^−/−^* mice do not develop a premature aging phenotype; however, several pathological changes can be observed in aged *Clock^−/−^* mice. The most dramatic phenotype developing with age in *Clock^−/−^* mice is cataracts. Cataract development is a major feature of aging in mammals; in this study, about 50% of wild type females and about 20% of wild type males developed cataracts with advanced age. *Clock^−/−^* mice developed cataracts with a higher frequency and significantly earlier than wild type mice: almost 100% of knockout animals had cataract with advanced age. We did not detect any defects in the lenses of young *Clock^−/−^* mice, which suggests that CLOCK is not necessary for lens development but instead is critical for normal lens physiology in adulthood. *Bmal1^−/−^* mice also develop cataracts, which can be a consequence of general accelerated aging in these mice. Development of cataracts upon CLOCK deficiency is more specific. Delay in cataract development in *Clock^−/−^* mice compared with *Bmal1^−/−^* mice can be explained by partial compensation of CLOCK function by NPAS2 or by the influence of multiple pathological changes in *Bmal1^−/−^* mice. Thus, both CLOCK and BMAL1 play an important role in lens physiology, strongly arguing that regulation of BMAL1:CLOCK transcriptional activity may be used for the prevention or treatment of age-dependent and possibly congenital cataracts.

Another major phenotype of *Clock^−/−^* mice was the increased frequency and severity of dermatitis. Skin inflammation is a common feature of aging in mice, and can be provoked by many factors including behavior, such as excessive grooming or fighting. CLOCK and BMAL1 are involved in the control of memory and adaptation to novelty [30] therefore, at this time we cannot exclude the possibility that increased dermatitis in *Clock^−/−^* mice is a consequence of changes in behavioral patterns; the detailed study of *Clock^−/−^* behavior is currently in progress. To dissect behavioral and non-behavioral components, mice of wild type and *Clock^−/−^* genotypes were caged together in random groups; however, dermatitis developed preferentially in *Clock^−/−^* mice, therefore, a behavioral component cannot be the only reason for dermatitis development. Interestingly, the circadian clock was recently implicated in the regulation of inflammatory response [31]; CLOCK deficiency significantly impairs circadian clock function in peripheral organs, thus, CLOCK may be involved in the regulation of skin inflammation through the circadian control of inflammation. CLOCK and BMAL1 proteins were recently implicated in the regulation of the hair cycle and hair follicle stem cell proliferation [32], thus, CLOCK deficiency can affect skin epithelium homeostasis, which also can contribute to the development of dermatitis. Although *Bmal1^−/−^* mice have a high frequency of corneal inflammation, we have never detected dermatitis in these mice. Presumably, these mice do not survive to the age at which dermatitis develops.

**Figure 6.**
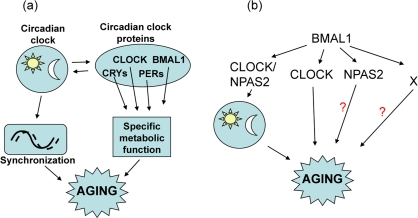
Model for the involvement of the circadian clock and circadian clock proteins in the control of aging. (**a**) The circadian clock synchronizes multiple metabolic processes in the organism and regulates the expression and activity of circadian clock proteins, which have unique physiological functions. Disruption of circadian clock functions will affect both activity and expression of circadian clock proteins and can contribute to aging; (**b**) BMAL1 can regulate aging through different pathways: through the circadian clock, through functional interactions with CLOCK, through interaction with NPAS2, and through circadian clock and CLOCK/NPAS2-independent mechanisms (indicated by X).

CLOCK deficiency also affects lifespan; the average lifespan of *Clock^−/−^* mice is reduced by 15% compared with wild type mice. CLOCK deficiency does not result in early mortality, and no significant differences in the death rate between genotypes at the beginning of survival curves was observed (Figure 1), which indicates that CLOCK deficiency does not result in the development of life-threatening specific pathologies at early or middle age. Nevertheless, the difference in the maximum lifespan between wild type and *Clock^−/−^* was dramatic, which suggests that CLOCK plays a more important role at advanced age. Together with the existing data on the lifespan of other circadian mutants, these data strongly argue for a functional connection between the circadian clock and aging. However, a deficiency of different clock components has different effects on longevity: BMAL1 deficiency results in a significantly reduced lifespan and multiple pathologies [9], a deficiency of CLOCK results in a milder phenotype, whereas the effects on longevity as a result of a deficiency of PERIODs [24] and a mutation of *Clock* [16] can be revealed only after challenge with irradiation. A growing literature on the pathological changes in circadian clock mutants suggests that the majority of the circadian clock proteins have unique physiological functions [4,33] which are not directly related to their functions in the circadian oscillator. Thus, deficiency of a specific circadian clock gene may contribute to the development of aging through two relatively independent mechanisms (Figure 6A). One mechanism is the effect of a circadian mutation on general clock functions such as synchronization of metabolic processes, and this effect is expected to be similar for all clock mutants. The second mechanism is the impairment of a specific function which is unique for a particular clock protein, and therefore the effect of a specific clock protein deficiency/mutation on aging will be a combination of these two mechanisms and will be unique for every circadian mutant.

The suggested hypothesis for the influence of the circadian proteins on aging may explain the difference in aging phenotypes of *Clock^−/−^* and *Bmal1^−/−^* mice. Indeed, BMAL1 regulates circadian rhythms through its interaction with CLOCK or NPAS2 (Figure 6B), therefore, a deficiency of BMAL1 or CLOCK eliminates the transcriptional activity of the BMAL1:CLOCK complex, but only BMAL1 deficiency disrupts circadian oscillations. The virtually normal lifespan of arrhythmic *Per1,2^−/−^* mice [24] suggests that the disruption of circadian oscillation *per se* does not have a significant effect on aging, therefore, accelerated aging of *Bmal1^−/−^* mice suggests circadian clock-independent functions of BMAL1 in regulation of aging. These clock-independent functions of BMAL1 may be associated with the BMAL1:CLOCK or BMAL1:NPAS2 transcriptional complex activities, but BMAL1 may have a unique (circadian clock- and CLOCK/NPAS2-independent) function in the control of aging. These “extracurricular” activities of BMAL1 on the one hand and circadian oscillations on the other may independently contribute to the regulation of aging; since BMAL1 deficiency affects both of these types of regulation, a BMAL1 deficiency has much stronger effect on aging than a deficiency of CLOCK.

The data obtained in this work suggest that CLOCK plays an important role in lens and skin homeostasis, as well as in regulation of mammalian lifespan. Further investigations on the aging of *Clock/Npas2* double-knock out mice and other circadian mutants will help to elucidate specific mechanisms and answer questions about the role of the circadian clock and individual clock components in aging.

## EXPERIMENTAL PROCEDURES

### Animals

*Bmal1^−/−^* mice were obtained from Dr. C. Bradfield (University of Wisconsin) [8], *Clock^−/−^* knockout mice were obtained from Dr. D. Weaver (University of Massachusetts Medical School, Worcester, MA) [19] details of target gene knockout strategies and animal generations can be found in the above cited publications. All mutants were backcrossed to C57BL/6J inbred strain (The Jackson Laboratory, Bar Harbor, ME, USA) for 10 generations. Wild type and knock out mice were generated by breeding of heterozygotic parents. Genotypes were determined using a PCR-based method as previously described [8,19]. Animals were maintained on a 12:12 light:dark cycle with lights on at 7:00 am, and fed on an 18% protein rodent diet (Harlan). 4 animals were housed per cage in a 29x18.5x12.7 cm (LxWxH) cage; if an animal died, the remaining cage-mates were maintained together. Cage beddings (corn cobs, Harlan) and nesting materials (pulped virgin cotton fiber, Ancare and aspen bedding, Harlan) were changed every week and cages were changed every two weeks. Animals were monitored by visual inspection daily and weighed weekly. Animals were sacrificed if they developed severe dermatitis or displayed any other signs of morbidity. All animal studies were conducted in accordance with the regulations of the Committee on Animal Care and Use at Cleveland State University.

### Cataract detection

Every two weeks animals were observed and opacity, which can be detected by gross examination, was scored. Each eye was scored independently. For statistical analysis, mice which died or were euthanized during the experiment have been treated as present before the time of death and as lost after time of death. The examinations were done independently by two examiners whom had no knowledge of animal genotype before examination.

### Dermatitis detection

Animals were observed daily and skin lesions which persisted for more than 1 week were counted as dermatitis after consultation with a veterinarian. Dermatitis was scored independently from the severity and body location, for example, one zone of dermatitis or several independent zones of dermatitis was scored as 1. If dermatitis resulted in formation of open skin wounds which persisted for 24 hrs, the animal was euthanized according to the regulations of the Committee on Animal Care and Use at Cleveland State University.

### Statistical analysis

39 *Clock^−/−^* mice (22 females and 17 males) and 38 wild type mice (20 females and 18 males) were used for the survival experiment (data are shown on Figure 2b). 15 *Clock^−/−^* mice (8 females and 7 males) and 12 wild type mice (7 females and 5 males) were euthanized due to severe dermatitis. Euthanized animals were excluded from analysis presented on Figure 2a. Data are shown as mean +/− standard deviation. The SigmaStat software package was used for analysis. The effects of genotype (circadian mutants versus wild type) on cataract development, dermatitis and lifespan were calculated with log rank statistical analysis. P<0.05 was considered as statistically significant.
